# Comparison of parental and practitioner’s acceptance for dental treatment under general anaesthesia in paediatric patients

**DOI:** 10.1186/s12887-022-03805-1

**Published:** 2023-01-28

**Authors:** Yassamin Djalali Talab, Margrit-Ann Geibel

**Affiliations:** 1 Danube Private University Krems, Krems an der Donau, Austria; 2 Department of Gender-Specific Dentistry, Danube Private University Krems, Krems an der Donau, Austria; 3 Department of Oral and Maxillofacial Surgery, Medical University of Ulm, Ulm, Germany

**Keywords:** Paediatric dentistry, General anaesthesia, Acceptance, Fear, Indications

## Abstract

**Background:**

Practitioner’s knowledge and parental perspectives on dental general anaesthesia (GA) have been surveyed separately in the past. But in daily routine both need to collaborate for the benefit of the child. The aim of this paper was to compare parental and practitioner’s acceptance of GA with special focus on identifying factors which influence their differences in decision making.

**Methods:**

Questionnaires were conducted among 142 participants in a specialized paediatric dental clinic in Germany from February 2020 to February 2021. 51 German practitioners from private practices and clinics participated. Data collection included: age, gender, experience with GA, fear of GA, risk evaluation and indications for GA.

**Results:**

There were no gender related differences in decision making. Emotional factors are present in parents of younger children. Parents are more likely to express fear and uncertainty regarding GA than dentists. Prior experience with GA significantly decreases fears in GA for parents. Both agree that extent of the treatment and low compliance are a suitable indication for GA. Dentists are more likely to accept GA due to a mental disability than parents. Parents were more likely to accept GA than dentists when multiple extractions were needed (regardless of compliance) or acute pain was present.

**Conclusions:**

A significant divergence in risk evaluation, acceptance and decision-making could be found in parents compared to dentists. Influencing factors are previous experience, younger age of the child, lack of knowledge and indication for GA.

**Supplementary Information:**

The online version contains supplementary material available at 10.1186/s12887-022-03805-1.

## Background

According to the German Association of Dental, Oral and Maxillofacial Surgery general anaesthesia (GA) for paediatric patients is a recommended treatment option when local anaesthesia cannot be administered due to low compliance, disabilities or medical conditions [16, 17]. Even though there has been constant improvement in oral health over the last decades [19] and a decline in birth rates, number of paediatric patients referral for GA has been constant [1].

Common complications for GA are postoperative nausea and vomiting (PONV), hypothermia, airway management complications, laryngo- or bronchospasm and pulmonary oedema [30]. Duration of GA have been linked to PONV and hypothermia. In Germany the mean duration of dental GA is 1,18 h, in Spain 2,25 h, in the US 55 min and in the UK 1,03 h [7–18]. However, current studies show that duration of GA does not influence PONV as significantly as thought [20, 32] and hypothermia occurs mostly in infant children younger than 1 year old which are not the target group for dental procedures. In general, only 0.5% of all dental paediatric GA show severe complications which makes GA a safe and routine procedure [8].

The warning of the U.S. Food and Drug Administration (FDA) from 2016 that the use of GA and sedation drugs in children younger than 3 years might affect the neural development has led to great medial attention and uncertainties [4, 21]. Current studies have shown that single brief exposures under 1 hour do not lead to neurocognitive deficits in children [27] and the factors inducing neurotoxicity are not conclusively determined [25, 31].

There are numerous studies investigating parental and practitioner’s perception of GA separately. Several studies showed that GA causes feelings of stress, fear and guilt in parents [2, 3]. Gender aspects also have been discussed in decision making [14]. Also, practitioner’s acceptance is related to their experience with GA and knowledge [27, 34].

However, in daily routine it is essential for parents and practitioners to collaborate for the benefit of the child. In this study we aimed to compare parental and practitioner’s acceptance of GA in Germany as no data relating this topic is currently available. Based on these results the doctor-patient relationship could be further improved.

## Methods

A cross-sectional study was conducted from February 2020 to February 2021 at a specialized paediatric clinic in Heinsberg, Germany. All the patients have been treated in this clinic.

Prior to the beginning of the study, questionnaires were distributed to 10 parents and 10 dentists to evaluate comprehensibility. Based on the outcome a minimum sample size of 142 participants was calculated. Only parents with children younger than 18 years were included. Parents who were unable to fill out the questionnaire due to language barriers were excluded from this study. Local German practitioners, from private practices and clinics were invited in context of paediatric dentistry conferences and through written invitation. Dental students, dentists without professional practice and orthodontists were excluded. They participated anonymously via online questionnaire due to local COVID-19 restrictions.

A written consent was obtained to collect anonymous data of the participants. The data collected was age and gender of parents/practitioner/child, three questions to prior experience of parents and dentists, two questions related to fear, three questions related to risk evaluation and 10 questions related to indications. Dentists were also asked in which field of dentistry they mainly work.

Questions were worded in German partially in a positive and negative manner to reduce the influence of wording in the decision making. Some questions were repeated in different phrasing to outline either objectional or emotional answers. The questionnaire was translated for publication purposes. Scores relating opinion could be given on a Likert scale of “I agree completely – I partially agree – I partially disagree – I disagree completely” and “I don’t know”. A factor (e.g. fear, prior experience) was seen as fulfilled when answered with “yes” or “I completely/partially agree”. Questionnaires for dentists were identical in regards of content but phrasing were changed when needed e.g. “my child” to “a child” (see additional files).

### Statistical analysis

The data was analysed and presented with IBM SPSS Version v.23.0 (IBM, Armonk, NY, US). Dichotomous answers were given value (1) for yes and (2) for no. Polytomous answers were given categorial values between one and four: (1) I agree completely, (2) I partially agree, (3) I partially disagree, (4) I disagree completely. The answer “I don’t know” or missing data were given the value 0. The mean is given with standard deviation (±SD) when appropriate.

At first descriptive analysis was conducted (histogram, bar chart) for an overview. To analyse significant correlations or differences statistical test were chosen according to the data structure (t-test, Spearman-test, χ2-test, Mann-Whitney-U-test). A *p*-value of 0.05 was chosen as significant.

## Results

### Demographic

The distribution of variables is shown in Table 1. The difference in mean age between parents (34.8 ± 10.5 years) and practitioners (39.9 ± 10.9) were non-significant (t-test, *p* = 0.25). The mean age of the children was 6.9 ± 4.2 years. Both in the parental (*p* < 0.00) and practitioner’s group (*p* = 0.01) women were predominant.

**Table 1:** Distribution of characteristics for dentists (*n* = 51), parents with experience (n = 51) and parents without experience (*n* = 40), χ2-test, * *p* < 0.05

Variables		Frequency n (%)	
**Gender**	**p**		
Dentists	0.01*	Male	16 (31.4)
		Female	35 (68.6)
Parents	0.000*	Male	10 (11)
		Female	81 (89)
Child	0.52	Male	54 (54)
		Female	46 (46)
**Age**			
Dentists		≤35 years	25 (49)
		> 35 years	26 (51)
Parents		≤35 years	42 (48.9)
		> 35 years	44 (51.1)
Child		≤3 years	23 (23)
		> 3 years	77 (77)
**Experience with GA**			
Dentists		Yes	45 (88.2)
		No	6 (11.8)
Parents		Yes	40 (44)
		No	51 (56)
**Occupation of dentists**			
Paediatirc dentistry		36 (64.3)	
General dentistry		14 (25)	
Oral and Maxillofacial Surgery		4 (7.1)	
Cosmetic dentistry		1 (1.8)	
Other		1 (1.8)	

### Risk evaluation and prior experience

Parents of small children had a higher risk evaluation when asked questions targeting an emotional response (Spearman-test, *p* = 0.02) but showed no difference to parents of older children when asked about permanent damage (Spearman-test, *p* = 0.10). The mean acceptance score for parents with children younger than 3 years old was 3.14 ± 0.53 and older children 2.8 ± 0.64. Age and gender of the parents did not have a significant influence on their risk evaluation (Mann-Whitney-U-Test, *p* = 0.10, Spearman-test, *p* = 0.68). The gender of the children did not influence parental decision-making (Mann-Whitney-U-Test, U = 53, *p* > 0.1).

Parental and practitioner’s answers to risk evaluation and fears are shown in Table 2. Parents without prior experience expressed more fears and concerns about risks than dentists. Surprisingly, over 50% of dentists completely or partially agreed to connect the thought of GA to risks while none of them thinks that GA leaves permanent damage (Table 2). Fear of dental GA negatively influenced the risk evaluation of all groups (Spearman, r = 0.471, *n* = 142, *p* < 0.00). Also, those parents/dentists that had higher scores in fear of GA showed significant less approval for GA indications like multiple extractions (not displayed, Spearman-test, *p* = 0.01) or non-compliance of the child (*p* = 0.01) compared to the rest of the cohort.

**Table 2:** Distribution of answers of parents with (w) or without (w/o) prior experience. Mann-Whitney-U-test, * p < 0.05

	Question	Group	I agree completely n (%)	I partially agree n (%)	I partially disagree n (%)	I disagree completely n (%)	I don’t know n (%)	p
**Risk evaluation**	**I think general anesthesia poses a low risk for complications.**	Parents w/o	8 (15.7)	22 (43.1)	18 (35.3)	3 (5.9)	0 (0)	0.003*
		Dentist	18 (35.3)	25 (49.0)	6 (11.8)	2 (3.9)	0 (0)	
								0.01*
		Parents w	5 (12.5)	23 (57.5)	9 (22.5)	3 (7.5)	0 (0)	
	**General anesthesia leaves permanent damage to the child.**	Parents w/o	0 (0)	8 (15.7)	31 (60.8)	9 (17.6)	3 (5.9)	0.000*
		Dentist	0 (0)	1 (2.0)	18 (35.3)	32 (62.7)	0 (0)	
								0.00*
		Parents w	0 (0)	5 (12.5)	26 (65.0)	9 (22.5)	0 (0)	
	**I connect the thought of general anesthesia to risks.**	Parents w/o	7 (13.7)	19 (37.3)	21 (41.2)	4 (7.8)	0 (0)	0.77
		Dentist	5 (9.8)	24 (47.1)	12 (23.5)	10 (19.6)	0 (0)	
								0.94
		Parents w	7 (17.5)	12 (30)	16 (40)	5 (12.5)	0 (0)	
**Fear**	**I am fearful to let my / a child be treated under GA**	Parents w/o	9 (17.6)	19 (37.3)	21 (41.2)	2 (3.9)	0 (0)	0.000*
		Dentist	1 (2)	18 (35.3)	12 (23.5)	20 (39.2)	0 (0)	
								0.13
		Parents w	3 (7.5)	11 (27.5)	21 (52.5)	5 (12.5)	0 (0)	
	**General anesthesia is a reasonable way to prevent dental phobia.**	Parents w/o	4 (7.8)	22 (43.1)	20 (39.2)	2 (3.9)	3 (5.9)	0.001*
		Dentist	27 (52.9)	14 (27.5)	5 (9.8)	5 (9.8)	0 (0)	
								0.33
		Parents w	15 (37.5)	18 (45.0)	4 (10)	3 (7.5)	0 (0)	

Prior parental experience with GA decreases fear (Mann-Whitney-U-Test, U = 764, *p* = 0.03) compared to parents without experience but does not decrease risk evaluation (U = 976, *p* = 0.58). As demonstrated in Fig. 2 self-perception of knowledge for experienced parents is significantly higher (U = 758, *p* = 0.03) compared to non-experienced parents but still lower than dentists (U = 550, *p* < 0.000). More knowledge in the practitioner’s group correlates with lower risk evaluation for permanent damage (not displayed, Spearman-test, *p* = 0.04), similar results were found in parents without experience (p < 0.00).

### Indications

A surgical procedure compared to a restorative treatment did not lead to more acceptance of GA in parents and dentists (*p* = 0.42). However, when parents were asked about “surgical frenectomy”, they showed significant approval for GA compared to practitioners (Table 3).

**Table 3:** Opinion on indications for GA (dentists *n* = 51, parents without experience n = 51), Mann-Whitney-U-test, * p < 0.05

Indication	Group	I agree completely n (%)	I partially agree ***n*** (%)	I partially disagree ***n*** (%)	I disagree completely ***n*** (%)	I don’t know ***n*** (%)	p	
**Surgical treatment**	Dentists	0	1 (2)	3 (5.9)	47 (92.2)	0		
	Parents	2 (3.9)	3(5.9)	18 (35.3)	28 (54.9)	0		
**Restorative treatment**	Parents	1 (2)	4 (7.8)	13 (25.5)	32 (62.7)	1(2)	0.42	0.317
	Dentists	0	1 (2)	2(3.9)	48 (94.1)	0		
**Frenectomy**	Parents	19 (37.3)	17 (33.3)	8 (15.7)	5 (9.8)	2 (3.9)	0.000*	
	Dentists	5 (9.8)	11 (21.6)	19 (37.3)	16 (31.4)	0		
**Low compliance due to fear**	Parents	16 (31.4)	22 (43.1)	10 (19.6)	3 (5.9)	0	0.05	
	Dentists	22 (43.1)	25 (49.0)	2 (3.9)	2 (3.9)	0		
**Mental disability**	Parents	12 (23.5)	19 (37.3)	11 (21.6)	7 (13.7)	2 (3.9)	0.000*	
	Dentists	35 (68.6)	12 (23.5)	4 (7.8)	0	0		
**Acute pain**	Parents	6 (11.8)	15 (29.4)	14 (27.5)	16 (31.4)	0	0.000*	
	Dentists	1 (2.0)	6 (11.8)	16 (31.4)	28 (54.9)	0		

Both groups show similar approval when treatment cannot be administered due to fear of the child (Table 3). Differences could be seen in mentally disabled paediatric patients: dentists showed more acceptance for GA (*p* < 0.00). Again, parents and dentists show different opinions about GA in case of acute pain: parent’s approval predominates (*p* < 0.00). No correlation between fear of GA to decision making for suitable indications for GA could be found (Spearman, *p* > 0.05).

**Fig. 1:**
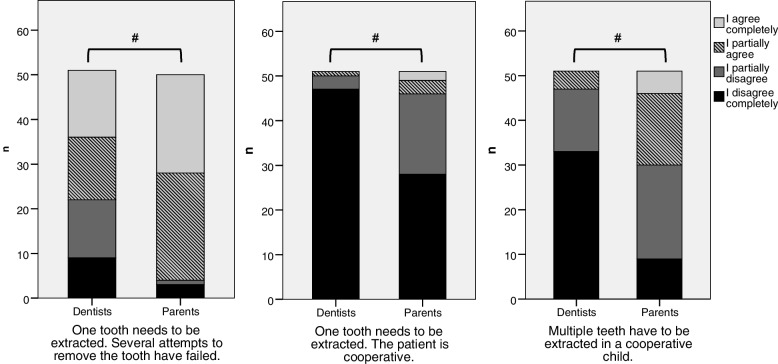
Comparison between dentists and parents’ approval of GA according to children compliance and number of extraction, Mann-Whitney-U-test,(*n* = 102), # *p* < 0.05, ns: non-significant

Evaluation of compliance and number of extractions can be seen in Fig. 1. Parents are more likely to approve of GA when multiple extractions (compared to single extraction) need to be done, even if the child is cooperative (Mann-Whitney-U-test, *p* < 0.00, not displayed). Similar to parents, dentists show a significant decrease in disapproval (not displayed, *p* = 0.01) when asked about multiple extractions; still their approval is significantly less pronounced compared to non-experienced parents (*p* < 0.00). Both groups agree that for single-tooth extraction the compliance of the child is a key factor (Mann-Whitney-U-test, *p* < 0.00).

**Fig. 2:**
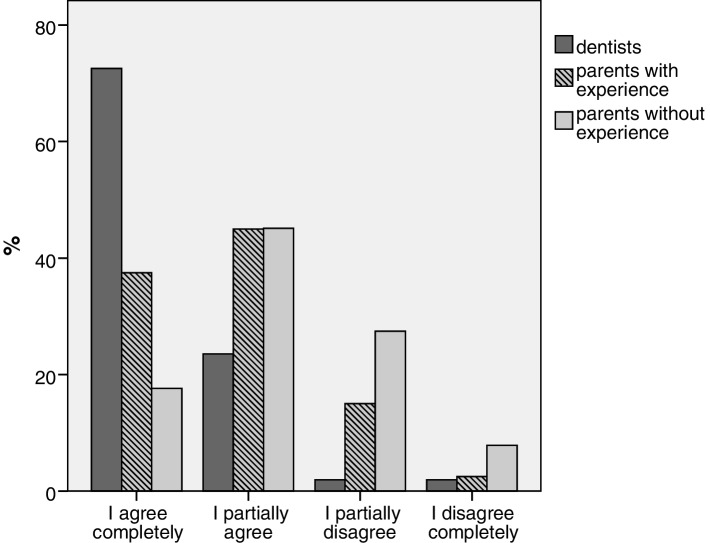
Comparison of the answers to „In my opinion I am well educated about the benefits and risks of general anaesthesia.“from parents with (*n* = 40) or without experience (*n* = 51) and dentists (*n* = 51)

## Discussion

To the author’s knowledge this is the first study to compare parental and practitioner’s acceptance of GA in Germany. As evidence-based data covering this subject is scarce, this study provides a source of evaluation and optimisation for the doctor-patient relationship. The study is not representative of Germany in statistical terms as the survey was not conducted on a national scale. Still, this study allows to get an insight on this poorly studied subject.

The study group was predominantly female. Every year the ratio of male to female practitioners in dentistry decreases in Germany (1,4:1 in 2010 vs. 1,2:1 in 2019) [12]. Paediatric dentists which were the majority of participating dentists are mostly female [26]. Surprisingly, the parent’s group was also mostly female, which highlights that in Germany domestic and medical concerns of the child are still mostly provided by the female caregiver [10]. The influence of parental gender has been discussed before. In line with our results Boka et al. [11] showed no influence in Greece while Chen et al. (2010) [14] showed the opposite for a Chinese population. A larger cohort with more male participants could give better understanding of their decision-making.

Emotional factors influencing parental perception of GA have been discussed in several studies [2, 3, 6], accordingly we found a significant relationship for parents of younger children (*p* = 0.02). Besides that, age of the child had no impact on their parents acceptance, similar results were shown by Chen et al. (2010) [14]. Keeping these opposing results in mind, we can conclude that subjective aspects of doctor-patient relationship like individual risk evaluation and perception of GA can be altered by emotions. At the same time, parents still might accept GA even though their individual perception is negative. Therefore, one should consider these possibilities and improve the parental and child’s experience by optimizing communication for a better overall outcome.

About 50% of the practitioners related GA to the thought of risks (Table 2). Uncertainty in practitioners can be due to lack of knowledge [27] or rare administration of GA [34]. Evaluating the actual experience of dentists by adding questions about the frequency of GA administered by the participants could provide a better insight. Negative media reports about GA after the warning of the FDA also have an impact [21]. To improve these uncertainties following guidelines and participating in training courses are a valid tool [28].

Prior experience to GA decreases fear in parents which Ohtawa et al. [24] has shown while their risk evaluation remains unaffected. As parent’s knowledge significantly correlates with their risk evaluation (*p* < 0.00) practitioners should focus on educating their patients in terms of likelihood of complications during or after GA.

In our study, there is consensus in both groups that low compliance and extent of treatment are an indication for GA which Campbell et al. 13 has reported before (2018) [13]. Also, both groups do not see a proper indication for GA when comparing a surgical to a restorative treatment (Table 3). However, when asked about surgical frenectomy parents preferred GA in comparison to dentists (*p* < 0.00) which might be due to the effect of medical terms usage for laypersons [15]. Therefore, language selection might influence parent’s perception and should be carefully chosen by the practitioner.

Higher acceptance of GA also could be seen for parents in case of acute pain (Table 3) and extended treatment (Fig. 1). Lack of knowledge of invasivity or alternative treatment options are possible reasons [29, 33]. In support of this conjecture, alternative treatment options like nitrous oxide inhalation sedation have been shown to be more accepted by parents than GA [11]. Sharing evidence-based recommendations and alternatives with the parents improves doctors-patient communication and their decision making.

Practitioners preferred GA when the patient had a mental disability compared to parents (Table 3). As the indication for GA is depended on the severity of disability [22, 23], it is questionable how much experience the parental group had with disabled children and as not specified what disability they meant. However, mentally disabled paediatric patients did not show higher complication rates which leaves GA as a reasonable and safe option [8, 24].

To further investigate these results, the questionnaire should be expanded on evaluation of fear of GA. Also, including factors like income, educational and social status of the participants could give more insight on this topic.

## Conclusions

As a conclusion, general anaesthesia is a topic connected to emotions, lack of knowledge and uncertainties which lead to different expectations. Differences in risk evaluations could be found for non-experienced parents and parents of younger children. Indications for GA like acute pain, disability and multiple extractions lead to diverging expectations. As a practitioner, it is key to educate oneself about the risks and complications as well as refer to guidelines and training courses.

## Supplementary Information

**Additional file 1.** Questionnaire for dentists: Translated questionnaire with scale answer options.

## Abbreviations

GA: general anaesthesia
FDA: U.S. Food and Drug Administration
PONV: postoperative nausea and vomiting
