# Supplementary figures and images for: Comparison of parental and practitioner’s acceptance for dental treatment under general anaesthesia in paediatric patients
Source: BMC Pediatr. 2023 Jan 28;23:45. doi: 10.1186/s12887-022-03805-1 (PMC9883120; doi:10.1186/s12887-022-03805-1)

## Slide 1
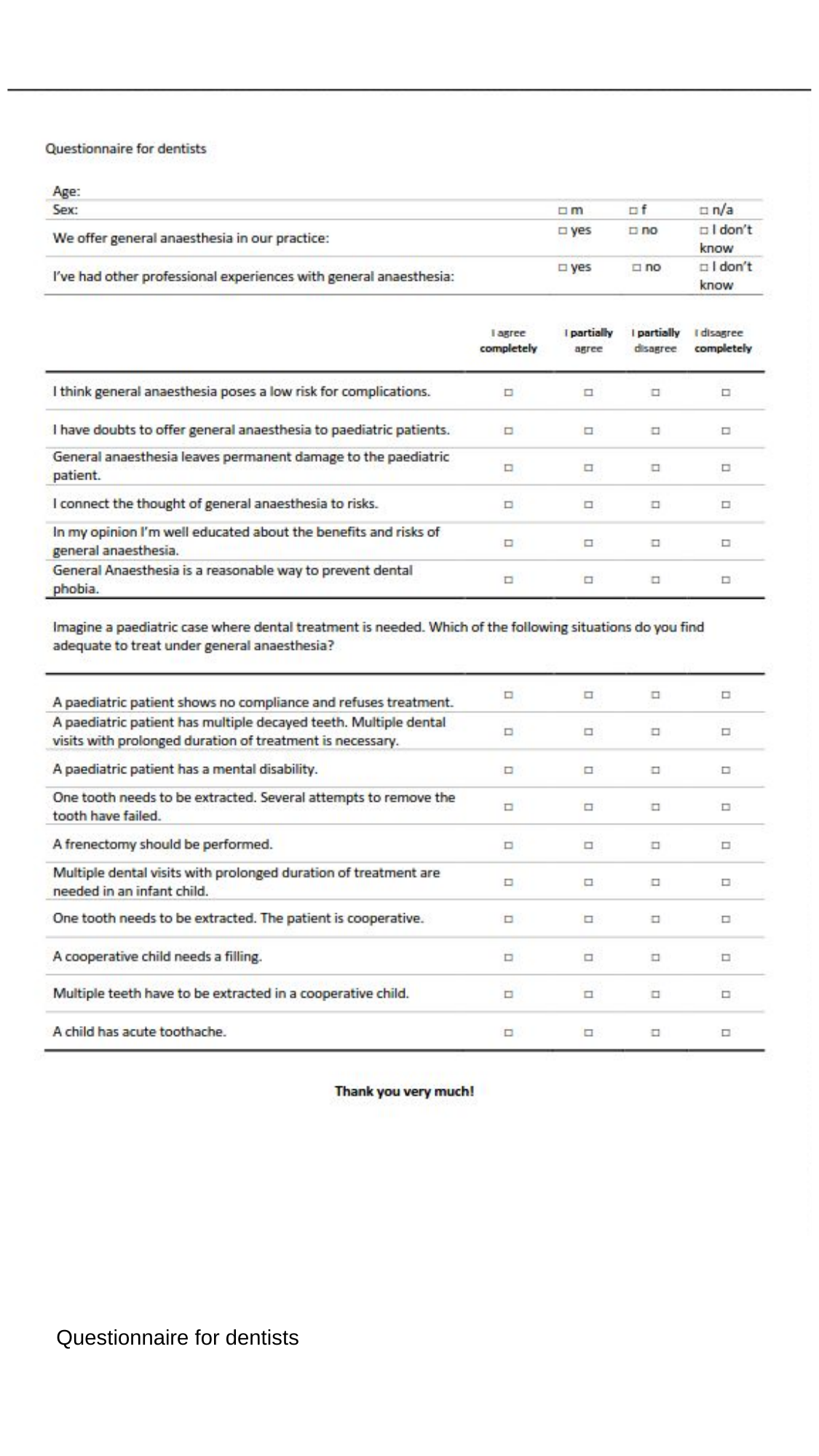

#
Questionnaire for dentists

Supplement: Supplementary file 1 — Additional file 1. Questionnaire for dentists: Translated questionnaire with scale answer options. [file 12887_2022_3805_MOESM1_ESM.pptx]
